# Symbolic time series analysis of electroencephalographic (EEG) epileptic seizure and brain dynamics with eye-open and eye-closed subjects during resting states

**DOI:** 10.1186/s40101-017-0136-8

**Published:** 2017-03-23

**Authors:** Lal Hussain, Wajid Aziz, Jalal S. Alowibdi, Nazneen Habib, Muhammad Rafique, Sharjil Saeed, Syed Zaki Hassan Kazmi

**Affiliations:** 1 University of Azad Jammu and Kashmir, Directorate of Quality Enhancement Cell, City Campus, Muzaffarabad, 13100 Azad Kashmir Pakistan; 2 Department of Computer Science, Faculty of Computing and IT, University of Jeddah, Jeddah, Kingdom of Saudi Arabia; 3 Department of Sociology, The University of Azad Jammu & Kashmir, Muzaffarabad, 13100 Azad Kashmir Pakistan; 4 Department of Physics, The University of Azad Jammu & Kashmir, Chehla Campus, Muzaffarabad, 13100 Azad Kashmir Pakistan; 5 Department of CS & IT, The University of Azad Jammu & Kashmir, City Campus, Muzaffarabad, Azad Kashmir Pakistan

**Keywords:** Symbolic dynamics, Physiological complexity, Normalized corrected Shannon entropy, Epileptic seizure, Interictal and ictal seizure states, Resting states, Brain activities

## Abstract

**Objective:**

Epilepsy is a neuronal disorder for which the electrical discharge in the brain is synchronized, abnormal and excessive. To detect the epileptic seizures and to analyse brain activities during different mental states, various methods in non-linear dynamics have been proposed. This study is an attempt to quantify the complexity of control and epileptic subject with and without seizure as well as to distinguish eye-open (EO) and eye-closed (EC) conditions using threshold-based symbolic entropy.

**Methods:**

The threshold-dependent symbolic entropy was applied to distinguish the healthy and epileptic subjects with seizure and seizure-free intervals (i.e. interictal and ictal) as well as to distinguish EO and EC conditions. The original time series data was converted into symbol sequences using quantization level, and word series of symbol sequences was generated using a word length of three or more. Then, normalized corrected Shannon entropy (NCSE) was computed to quantify the complexity. The NCSE values were not following the normal distribution, and the non-parametric Mann–Whitney–Wilcoxon (MWW) test was used to find significant differences among various groups at 0.05 significance level. The values of NCSE were presented in a form of topographic maps to show significant brain regions during EC and EO conditions. The results of the study were compared to those of the multiscale entropy (MSE).

**Results:**

The results indicated that the dynamics of healthy subjects are more complex compared to epileptic subjects (during seizure and seizure-free intervals) in both EO and EC conditions. The comparison of the dynamics of epileptic subjects revealed that seizure-free intervals are more complex than seizure intervals. The dynamics of healthy subjects during EO conditions are more complex compared to those during EC conditions. Further, the results clearly demonstrated that threshold-dependent symbolic entropy outperform MSE in distinguishing different physiological and pathological conditions.

**Conclusion:**

The threshold symbolic entropy has provided improved accuracy in quantifying the dynamics of healthy and epileptic subjects during EC an EO conditions for each electrode compared to the MSE.

## Introduction

Epilepsy is monitored using electroencephalography signals (EEGs) and epileptic seizure detection algorithms [1]. About 50 million people across the globe are suffering from epilepsy [2], including patients of all age groups from newborns [3] to senior adults [4]. The behaviour, cognitive functions and mood of epileptic patients affect the epileptic activities within the brain. Moreover, patient’s psychological and social adaptation can be modified according to their epileptic experience. Due to the interactions between these aspects, people who suffered from epilepsy may face many psychological and cultural problems [1, 5–9].

Various techniques have been developed for understanding the mechanism of epileptic disorders and epileptic seizure detection [10–12] based on time–frequency decomposition [13] and wavelet-based spare functional linear model [14]. Alkan and Kiymik [15] used AR and Welch methods for detection of epileptic seizure and by examining the power spectra and power spectral densities. Buteneers et al. [16] used reservoir computing (RC) to detect epileptic seizure on intercranial rate data. Bogaarts et al. [17] employed a support vector machine (SVM) to classify and optimize neonatal EEG seizure detection by first filtering the EEG features and data using Kalman filter (KF) in order to increase the temporal precision. Fergus et al. [18] used an advanced machine learning approach on generalized epileptic seizure detection of CHB-MIT database. Recently, researchers have employed DWT-based ApEn and artificial neural network [19], probability distribution based on equal frequency discretization [20], and best basis wavelet functions in temporal lobe mimetic [21] for detection and analysis of EEG epileptic seizures.

The non-linear dynamics in normal resting-state EEG are primarily concerned with studying the dynamics in normal EEG particularly in alpha rhythm. Generally, alpha activity in EEG is dominant in normal individuals during an eye-closed resting condition and suppresses as visual stimulation [22–25]. Alpha activity decreased in occipital regions and also in posterior regions when the individuals opened their eyes [26–30]. These studies suggest that alpha desynchronization is reflecting the increased visual system functioning due to visual stimulation being mediated by the reticular activating system [23, 25]. Alpha rhythm biofeedback has gotten some successes in humans for seizure suppression and for depression treatment [26]. Aich [31] examined the relationship between epilepsy, seizure activity and alpha activity in EEG. The findings revealed that the absence of alpha rhythm activity is significantly correlated with the presence of seizure activity. Sherman et al. [32] tracked thalamo-cortical association during pentylenetetrazol (PTZ) seizures in rats with and without prior treatment with anti-epileptic drug (AED) that raises the threshold for seizure. The findings revealed the formation of non-linearities at specific frequencies in the recorded EEG due to the increase in low alpha wave harmonic AED therapy.

Previous research evidences reported that physiological systems operate across multiple temporal and spatial scales [33–36]. Traditional entropy-based methods such as approximate entropy [37] and sample entropy [38] which are single-scale-based models quantify the complexity of physiological systems by computing the repetitive patterns of a time series to quantify the degree of its regularity. To address this issue, multiscale entropy (MSE) was proposed [33] which incorporates multiple time scale accurate entropy estimate. Kang et al*.* [39] used MSE to track and differentiate dynamical changes in complexity of each sub-band under hypothermia and normothermia conditions. Park et al. [40] demonstrated that dynamical alternations owing to Alzheimer disease can be effectively described by MSE curves. In a study conducted by Ouyang et al. [41], dynamical characteristics during seizure-free, pre-seizure and seizure states in epileptic rates were investigated. The results revealed that the dynamics of seizure-free state are more complex than pre-seizure and during seizure state dynamics.

The methods derived from symbolic dynamics provide another framework to deal with the underlying multiscale character of physiological systems and could improve the accuracy in quantifying the dynamics of health and epileptic subjects. Aziz and Arif [35] proposed threshold-dependent symbolic entropy for distinguishing control (healthy) and neurodegenerative disease patients (ALS, Huntington and Parkinson). The symbolic time series was also used to analyse the temporal gait dynamics of human locomotor system during constrained and metronomically walking protocols and observed that the output of locomotor system during unconstrained normal walking are more complex than slow, fast or metronomically paced walking [42].

The presented study was conducted to determine the dynamics of healthy and epileptic seizure subjects as well as to discriminate eye-closed (EC) and eye-open (EO) states during resting conditions, using threshold-dependent symbolic entropy. The threshold-based symbolic entropy was employed to distinguish EEG healthy (both with the eyes open and closed) and EEG epileptic seizure subjects (epileptic subjects, i.e. interictal during seizure-free interval; epileptic subjects, i.e. ictal during seizure interval). The normalized corrected Shannon entropy (NCSE) [31] was used to quantify dynamics, the complexity of healthy (set O and set Z) subjects and epileptic (set F, set N and set S) subjects at different threshold values as well as to discriminate EC from EO during resting states at various thresholds. The results of the study were compared to those of the MSE [33].

## Methods

### EEG datasets

The performance of threshold-dependent symbolic entropy and MSE was evaluated using two different datasets. The first dataset was taken from a publicly available database (http://epileptologie-bonn.de/cms/front_content.php?idcat=193&lang=3&changelang=3), made available by the Department of Epileptology, Bonn University, and its detailed description is provided by Andrzejak et al. [43]. The EEG data was recorded with 128-channel amplifier system, using an average common reference. The data were digitized at a sampling frequency of 173.61 Hz using a 12-b resolution. The spectral bandwidth of the data acquisition system varied from 0.5 to 85 Hz. The whole EEG data comprised of five sets (denoted as Z, O, N, F and S), each containing 23.6-s-duration 100 single-channel EEG segments. The sets O and Z were recorded from five healthy volunteers during awake state with the eyes closed (set O) and the eyes open (set Z) using a standardized electrode placement scheme. Sets N, F and S originated from an EEG archive of pre-surgical diagnosis. Segments in set F were acquired from the epileptogenic zone and those in set N were acquired from the hippocampal formation of the opposite hemisphere of the brain. The segments in sets N and F contained EEG recordings acquired during seizure-free intervals, and segments in set S contained seizure activity.

The second dataset comprised of EEG recordings acquired from 16 healthy (6 males and 10 females) subjects during EC and EO conditions in resting state, and this data has been used in various studies [44–47]. The EEG data is publically available at (https://www.nbtwiki.net/) and has been used as demo dataset for Neurophysiological Biomarker Toolbox. Two versions, raw and clean EEGs, are available, and in the present study, the clean version of data is used. All volunteers refrained from consuming caffeine, alcohol, and nicotine for at least 6 h before the experiment. The EEG was recorded outside the MRI scanner using two BrainAmp amplifiers (Brain Products, Munich, Germany) connected to the EEG monitor via optical fibres [47]. The EEG was recorded in DC mode at a resolution of 0.1 V, with a 250-Hz low-pass filter [47]. The standard 10–20 positions of the electrodes were used as reported by Halder et al. [44], Maurer et al. [45], and Brem et al. [46]. A 2.5-min EC resting-state EEG recordings at a resolution of 3.3 mV with a sampling frequency of 5000 Hz were acquired. These recording were downsampled to 500 Hz offline [47]. The subjects were instructed to relax and stay on the scanner while remaining calm and awake. For both resting conditions, i.e. EC and EO, the subjects were additionally instructed accordingly. The EEG datasets with EC and EO were pre-processed offline using Brain Vision Analyzer software (version 1.05, brain Products, Munich, Germany) and the Neurophysiological Biomarker Toolbox (http://www.nbtwiki.net/). Major large muscles or motion artefacts (typical duration 2.5 s, range 0.4–31.6 s) were removed from the EEG before ICA. Moreover, data were digitally high-pass filtered for ICA cleaning (0.5-Hz finite impulse response filter, 4-s Hanning window).

### Threshold-dependent symbolic entropy

The symbolic time analysis involves the transformation of the original time series into a series of discretized symbols that are processed to extract useful information about the state of the system generating the process. There have been many recent applications of symbolic analysis for biological systems. The NCSE is calculated for discretized symbols. This method requires the following steps: while using the symbolic time series analysis, most of the microscopic detail of dynamics may be lost; however, the temporal dynamics are embedded in the structure according to length—*L* distribution [20, 48–50]. The symbolic time analysis is efficient in terms of computer time and storage and noise robustness.

#### Symbolization process

The EEG time series of both epileptic seizure and eye-open and eye-closed conditions in resting states are transformed into symbol sequence and word sequence by following the procedure detailed below [35, 51].

Step I:

Consider an EEG time series *T* = {*T*_*i*_, *i* = 1, 2, … … …. *N*}. The time series is transformed into symbol sequence as *t* ^Ψ^ = {*t*_*i*_^Ψ^, *i* = 1, 2, …. *N*} having fixed values of quantization level (*psi* Ψ) labelled with number zero to Ψ − 1. Each sample of time series is normalized, and we obtained a time series *t* = {*t*_*i*_, *i* = 1, 2, … … …. *N*}.

We used the quantization level 2 (symbol 0 and 1) according to the following symbolization criteria:

1 $$ {s}_i=\left\{\begin{array}{c}\hfill 1\ \mathrm{if}\kern0.5em {t}_i\ge \theta \hfill \\ {}\hfill \kern1.5em 0,\ \mathrm{otherwise}\hfill \end{array}\right. $$

where *θ* is the threshold and is computed as the mean of the EEG time series.

Consider, for example, EEG time series of an EC resting state taking only 10 data points to illustrate the symbolization process such as the following:

*T* = {0.5378, 2.0403, 5.1684, 7.8292, 7.3310, 3.4433, 0.4669, −0.3348, −0.7980, −2.2799}, where threshold (*θ*) is $\overline{t}\ \left(\mathrm{mean}\ \mathrm{of}\ \mathrm{time}\ \mathrm{series}\right)=2.3404$

**Fig. 1:**
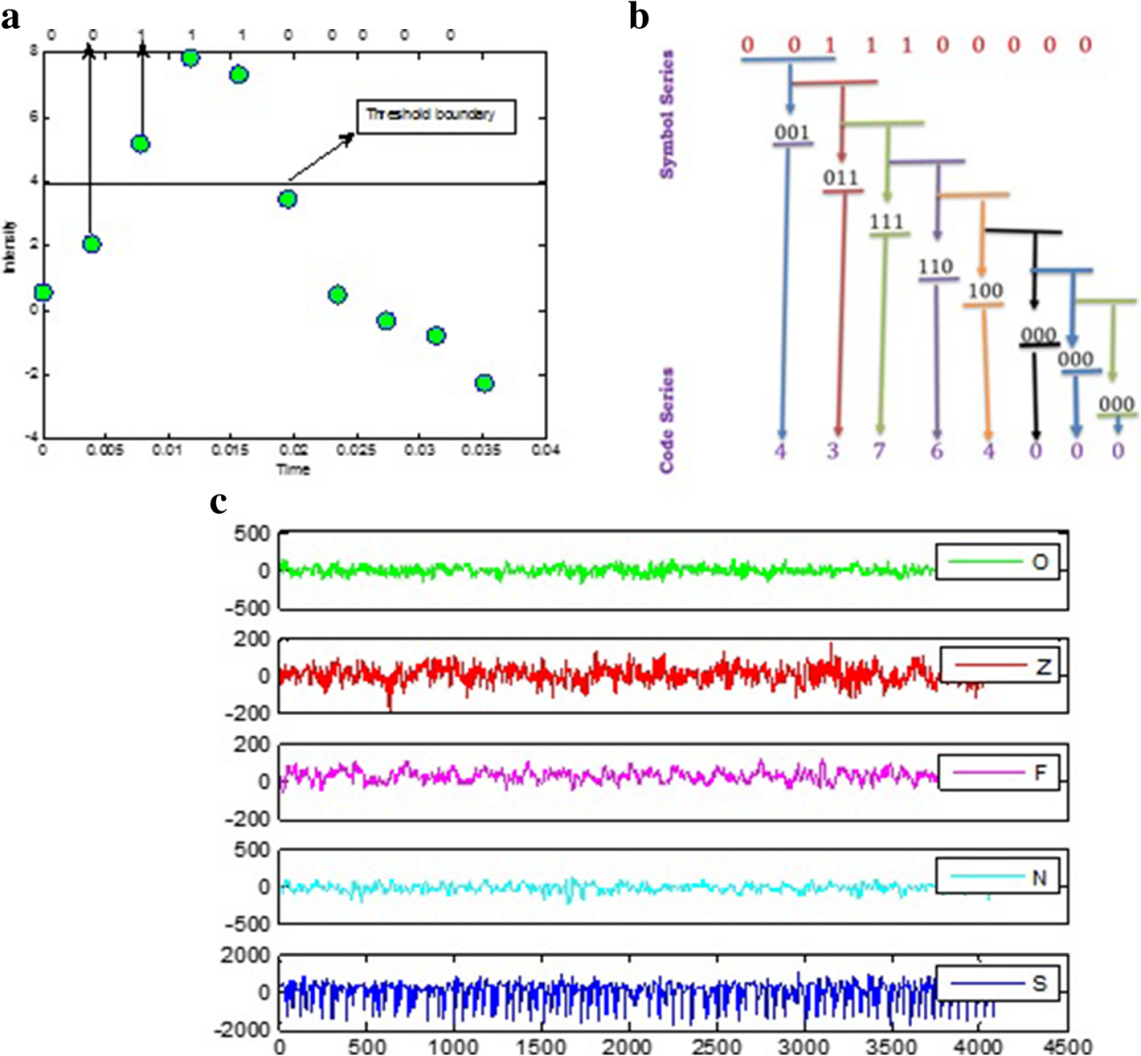
Symbolic time series analysis and EEG time series plots of five datasets. **a** EEG time series separated by threshold and conversion into binary series. **b** Data symbolization process. **c** Exemplary EEG time series from each of the five sets. From *top* to *bottom*: set O to set S (denoted EEG-O to EEG-S). Amplitudes of the surface EEG recordings are typically in the order of some μV. For intracranial EEG recordings, amplitudes range around some 100 μV. For seizure activity, these voltages can accede 1000 μV

Symbol series using Eq. (1) was formed as {0 0 1 1 1 0 0 0 0 0 0 } as shown in Fig. 1a, b.

Step II:

The word sequence is formed by dividing the symbol sequence of a word length *L* of three or more words. To construct the symbol sequence (words) from symbol series in step I, the group of symbols are collected together in temporal order by moving the symbol series one step at a time, where each step reveals a new sequence using:

2 $$ {t}_{L, i}^{\Psi}=\left\{{t}_i^{\Psi},{t}_{i-1}^{\Psi},\dots .{t}_{i- L+1}^{\Psi}\right\} $$

The following word series is generated from the symbol series generated using Eq. (2) taking word length *L* = 3.

$$ \mathrm{Symbol}\ \mathrm{series}=\left\{(001),(011),(111),(110),(100),(000),(000),(000)\right\} $$

Step III:

Each possible sequence generated by step II is represented in terms of a unique identifier; this new time series is referred to as a code series [13] using the following procedure:

3 $$ {w}_i=\left\{{t}_i^{\Psi}.{\Psi}^{L-1},{t}_{i-1}^{\Psi}.{\Psi}^{L-2},\dots \dots \dots {t}_{i- L+1}^{\Psi}.{\Psi}^0\right\} $$

Finally, the code series are as follows:

$$ \mathrm{Code}\ \mathrm{series}=\left\{1\ 3\ 7\ 6\ 4\ 0\ 0\ 0\right\} $$

To unfold the dynamics of time series, word length plays a vital role as described by [32]. They also pointed out that the optimal word length correlates with the number of data points in the time series and by increasing the word length, NCSE decreases. We also observed that NCSE decreases by increasing the word length [35], and the optimal results are obtained using world length *L* = 3 in this study.

#### Symbolic sequence analysis

Data symbolization can be done with several methods (e.g. heart rate variability using two bins), the value ‘1’ or ‘0’ is assigned to each bin according to its occurrence [50] as described in the below example [52, 53]. This is usually done by replacing the original data with relative symbols according to the threshold criterion as described in [35]. Moreover, Cysarz et al. [51] quantified the dynamics in a heart rate using different symbolic dynamics binning approaches. Symbolic dynamics have been widely used to quantify the non-linear dynamics of physiological time series. There are several approaches to symbolize the time series, e.g. one approach is to symbolize the time series using deviation from an average heart rate as used by Kurths et al. [54] and Wessel et al. [55]. After obtaining the symbol patterns, further complexity can be analysed using Shannon or Renyi entropy [35, 51]. The non-linear dynamics have also been investigated using time–frequency methods such as correlation, coherence, and interaction and synchronization measures. The basic symbolization approach is also extended to the time-delayed coupling methods in HRV and EEG as described in [56].

Symbol sequence analysis depends on quantitative measures of symbol sequence frequencies. Classical theory (containing examples from Euclidean norm and chi-square statistics) and information theory (containing examples from Shannon and generalized Renyi entropy) are the two general divisions of symbolic sequence analysis. In this study, we have followed the information theoretic approach using Shannon entropy.

1. Shannon entropy
  Shannon entropy of order *L* and quantization level Ψ can be defined as
  4 $$ \mathrm{S}\mathrm{E}\left( L,\Psi \right)=-{\displaystyle \sum } p\left({t}_L^{\Psi}\right) \cdot { \log}_2 p\left({t}_L^{\Psi}\right) $$
  where *p*(*t*_*L*_^Ψ^) is the probability of the time series *t*_*L*_^Ψ^ being the pattern and Ψ is the quantization level labelled with number from zero to Ψ − 1. Eguia et al. [57] reported that systematic error or bias and random errors affect the estimates computed by using Shannon entropy and thus reported a leading correction of entropy.
2. Corrected Shannon entropy
  The corrected Shannon entropy is defined as
  5 $$ \mathrm{C}\mathrm{S}\mathrm{E}\left( L,\Psi \right)=\mathrm{S}\mathrm{E}\left( L,\Psi \right)+\frac{C_R-1}{2 M \ln 2} $$
  where M is the total number of words and *C*_*R*_ is the number of words which occurred among the possible outcomes of words. For certain quantization level Ψ and word length *L*, the value of CSE is the maximum, and when all words M occur uniformly, thus, max CSE will be:
  6 $$ {\mathrm{CSE}}^{\max}\left( L,\Psi \right)=-{ \log}_2\left(\frac{1}{M}\right) + \frac{M-1}{2 M \ln 2} $$
  From Eq. (6), it can be observed clearly that the maximum value of CSE cannot be the same for two different word lengths and when *L* increases, *M* also increases thereby increasing the maximum value of CSE. Likewise, it is not possible to compare two values of CSE having different word lengths at the same threshold and quantization level. To overcome this problem, NCSE is proposed and used by Aziz and Arif [35].
3. Normalized corrected Shannon entropy
  Thus, NCSE can be defined as
  7 $$ \mathrm{NCSE}\left( L,\Psi \right)=\frac{\mathrm{CSE}\left( L,\Psi \right)}{{\mathrm{CSE}}^{\max}\left( L,\Psi \right)}=\frac{\mathrm{CSE}\left( L,\Psi \right)}{-{ \log}_2\left(\frac{1}{M}\right) + \frac{M-1}{2 M \ln 2}} $$
  Using Eq. (7) for any quantization level (Ψ) and word length (*L*), the value of NCSE will always vary from 0 to 1.

#### Statistical analysis

The NCSE values were not following the normal distribution, and Mann–Whitney–Wilcoxon (MWW) test was used to find significant differences among various groups at 0.05 significance level. The MWW is a non-parametric analogue of *t* test, which does not assume any properties regarding the distribution of the independent variable. All the codes for the topographic maps and statistical and symbolic time series analysis were developed in Matlab.

## Results

NCSE was employed to distinguish the EEG healthy (set O and set Z) with epileptic subjects (interictal and ictal intervals) and eye-closed resting (ECR) and eye-open resting (EOR) states. The mean ± SD against each group are shown in Tables 1 and 2. The results show that the NCSE value of the healthy EEG subjects is higher than that of the epileptic subjects (both with seizure and seizure-free intervals) as shown in Table 1 as well as the NCSE value of the EO condition than that of the EC condition during resting states in Table 2 at a smaller threshold range. The results also reveal that an epileptic seizure-free interval, i.e. interictal (F and N), has higher complexity than the epileptic with seizure (set S) at the same smaller threshold values. The maximum NCSE value for the healthy subjects (with eye-open set O) was found at a threshold of 30 ms, whereas the maximum threshold for the healthy subject (with eye-closed set Z), epileptic seizure-free intervals (interictal intervals) was found at a threshold of 25 ms; however, the NCSE value for epileptic seizure (ictal interval) increases until a threshold of 80 ms.

**Table 1:** Values of NCSE mean ± SD set O (healthy with the eyes open) with S, F and N at various threshold levels

(*θ*)	Set O	Set Z	Set S	Set F	Set N
	Mean ± SD	Mean ± SD	Mean ± SD	Mean ± SD	Mean ± SD
15	0.74 ± 0.01	0.80 ± 0.00	0.37 ± 0.01	0.66 ± 0.01	0.67 ± 0.01
20	0.80 ± 0.01	0.83 ± 0.00	0.42 ± 0.01	0.68 ± 0.01	0.69 ± 0.01
25	0.83 ± 0.01	0.83 ± 0.00	0.45 ± 0.01	0.68 ± 0.01	0.69 ± 0.01
30	0.84 ± 0.00	0.80 ± 0.01	0.49 ± 0.01	0.66 ± 0.01	0.68 ± 0.01
35	0.83 ± 0.00	0.76 ± 0.01	0.51 ± 0.01	0.64 ± 0.01	0.65 ± 0.01
40	0.82 ± 0.01	0.71 ± 0.01	0.53 ± 0.01	0.62 ± 0.01	0.63 ± 0.01
45	0.80 ± 0.01	0.66 ± 0.01	0.56 ± 0.01	0.58 ± 0.01	0.60 ± 0.01
46	0.79 ± 0.01	0.65 ± 0.01	0.56 ± 0.01	0.58 ± 0.01	0.59 ± 0.01
50	0.77 ± 0.01	0.61 ± 0.01	0.58 ± 0.01	0.56 ± 0.01	0.57 ± 0.02

*O* (eyes open) healthy subjects data, *Z* (eyes closed) healthy subjects data, *N* and *F* epileptic subjects during seizure-free interval (interictal), and *S* epileptic subjects during seizure interval (ictal)

**Table 2:** Maximum median values of NCSE with the maximum significance level from threshold ranges from 1 to 30 and MSE for scale 1 to 20 using non-parametric Wilcoxon rank sum test

Electrode	Symbolic time series					MSE				
	Max. value of NCSE		Minimum *P* value (significance)		Significance (*θ*, 1–30)	Max. value of MSE		Minimum *P* value (significance)		Significance (scale, 1–20)
	EC	EO	*P* value	Threshold	Threshold	EC	EO	*P* value	Scale	Scale
C3	0.83	0.85	**0.704	1.2	1–1.2	2.40	2.42	*0.364	6	6
C4	0.83	0.85	*0.478	1	1	2.41	2.43	NS	NA	NA
Cz	0.82	0.85	*0.122	1.2	1–2	2.41	2.44	*0.150	7	4–7, 19
F3	0.83	0.86	**0.145	1.4	1–1.2; 4–10	2.41	2.42	*0.150	7	1–2, 6–7
F4	0.84	0.86	***0.430	1.4	1–2; 4–20	2.41	2.43	*0.109	6	1, 6
F7	0.84	0.85	***0.490	1.2	1–1.4; 4–10	2.42	2.42	*0.167	6	6
F8	0.84	0.86	**0.700	1.2	1–1.4	2.39	2.40	NS	NA	NA
Fp1	0.84	0.87	*0.122	1.4	1–2; 9–10	2.38	2.39	NS	NA	NA
Fp2	0.84	0.86	NS	NS	NS	2.40	2.38	NS	NA	NA
Fz	0.83	0.86	**0.273	6	1–1.4; 3.6–20	2.41	2.43	NS	NA	NA
O1	0.84	0.86	***0.130	1.4	1–3; 5–30	2.39	2.42	*0.478	10	10
O2	0.83	0.85	***0.430	1–1.2	1–2; 3.6–30	2.39	2.42	*0.364	20	9–11, 20
P3	0.84	0.85	**0.443	1–1.2	1–1.4; 3.6–9	2.42	2.44	NS	NA	NA
P4	0.83	0.84	**0.349	1.2	1–1.4; 4–15	2.42	2.44	*0.226	6	5–6
Pz	0.82	0.84	*0.122	1.4	1–2; 5–7	2.38	2.42	*0.437	19	19
T7	0.83	0.85	**0.983	1.4	1–1.4; 4.4–6.5	2.42	2.42	NS	NA	NA
T8	0.84	0.86	*0.302	1.4	1–1.4	2.41	2.43	*0.150	1	1
P7	0.84	0.85	***0.320	1	1–1.4; 3–25	2.41	2.43	NS	NA	NA
P8	0.83	0.84	***0.850	1.2	1–2; 4–30	2.42	2.44	*0.437	1	1

*NS* not significant, *NA* not applicable

***Strictly significant, *p* < 0.0001; **very significant, 0.001 <=*p* < 0.01; *just significant, 0.01 <=*p* < 0.05; ^~^almost significant, 0.05 <=*p* < 0.1

The p-value results indicated in italicized using threshold based symbolic entropy are more significant than MSE results at each electrode

The NCSE values are computed from a threshold of 1 to 30 ms in which the significance results are obtained overall from both eye-closed and eye-open conditions during resting states for 19 channels C3, C4, Cz, F3, F4, F7, F8, Fp1, Fp2, Fz, O1, O2, P3, P4, Pz, T7, T8, P7 and P8 according to 10–20 system. MSE was also estimated with *m* = 1 and *r* = 0.25 times the SD of the original time series. The results are summarized in Table 2. The values of EO for NCSE and MSE at all electrodes are higher than those of EC. These electrodes are chosen according to 10–20 international standard system and are representing the overall 129 other electrodes as usually used in the research to investigate the non-linear dynamics such as correlation, coherence, complexity or any spectral measure.

**Fig. 2:**
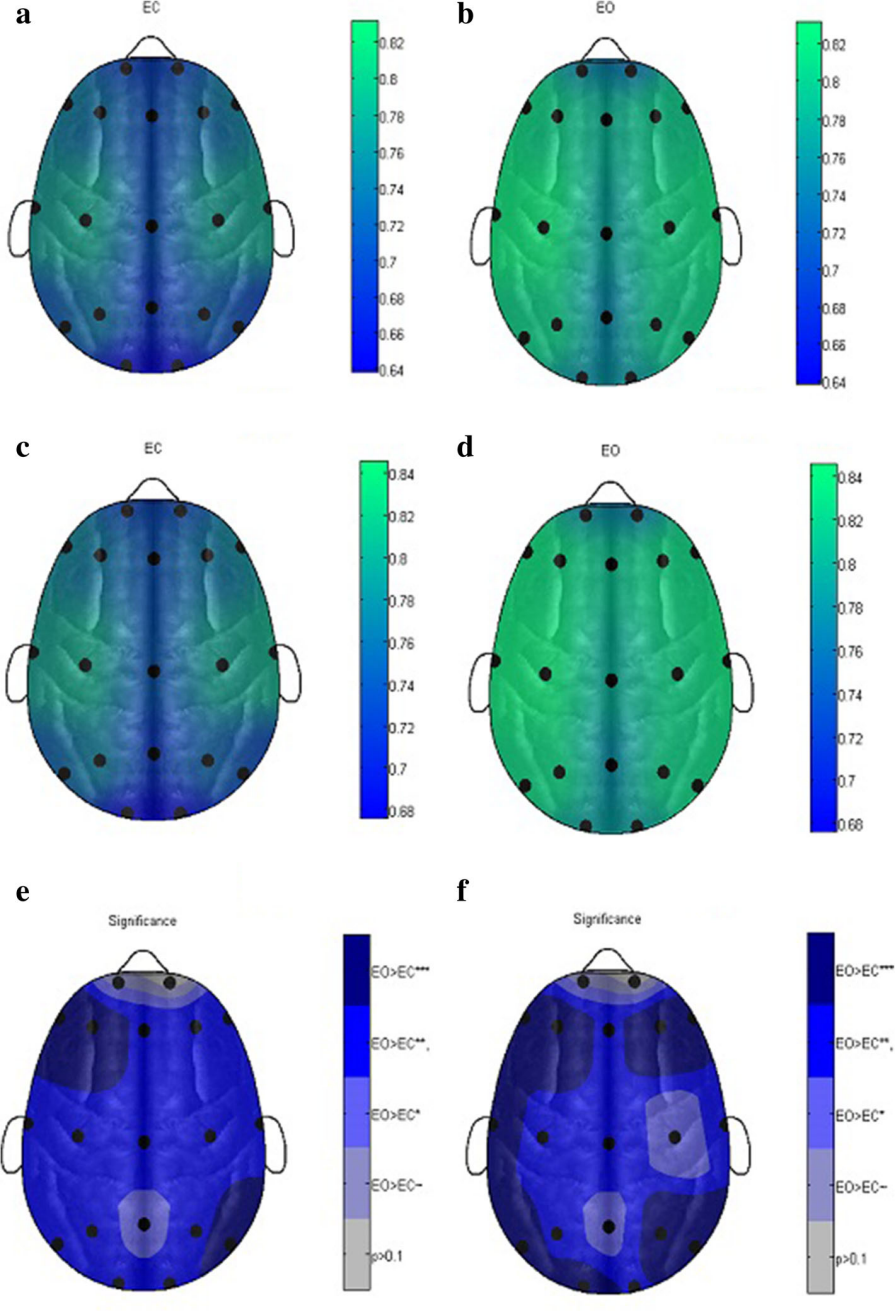
Topographic map representation of 19 electrodes according to standard 10–20 system with median NCSE values. **a** EEG eye-closed subject during a resting state at threshold 1.2 and word length of 3. **b** EEG eye-open subject during a resting state at threshold 1.2 and a word length of 3. **c** EEG eye-closed subject during a resting state at threshold 1.4 and a word length of 3. **d** EEG eye-open subject during a resting state at threshold 1.4 and a word length of 3. **e** EEG paired-wised significance EC vs EO at threshold 1.2 and a word length of 3. **f** EEG paired-wised significance EC vs EO at threshold 1.4 and a word length of 3

Table 2 reflects the median NCSE values of EC and EO during resting states with maximum separation, i.e. significance values in the threshold range 1 to 30 using non-parametric Wilcoxon rank sum test, and results are compared to MSE with a time scale from 1 to 20. The maximum medium NCSE values with the highest separation (minimum *P* value) significance value for EC and EO during resting states are reflected in Table 2 with a corresponding threshold also reflected where these highest significance levels are obtained. The column Significance (*θ*, 1–30 ms) in Table 2 reflects the threshold ranges in which the statistically significance was obtained from threshold from 1 to 30 ms. The results show that 18 electrodes out of 19 selected standard electrodes exhibit the significant results in the threshold range 1 to 30, and only Fp2 electrodes did not show any significant result on any threshold. From the results, it is also evident that the highest significance level at all electrodes was obtained at thresholds 1.2 and 1.4 in most of the cases and at thresholds 1 and 6 in few cases. The results also reflect that NCSE gives significant results at frontal electrodes in threshold range 1–2 and 4–10 in most of the cases whereas the occipital and parietal region electrodes exhibit significance in lower range 1–2 and upper range 4–30 thresholds. The central electrodes C3, C4 and Cz give significant results at smaller threshold values in the range 1–2 only whereas central, occipital and parietal probes exhibit these significances even at higher thresholds from 4 to 15/30. Overall, NCSE gives the highest significant results in all the regions such as frontal, central, parietal, occipital and temporal regions using 19 selected electrodes as per standard in the threshold range 1–30. The maximum median values of the NCSE values and corresponding significance values for both EO and EC are reflected in the form of topographic maps in Fig. 2a–d at thresholds 1.2 and 1.4 and a world length of 3. The MSE values for EC and EO are also quantified for scale 1 to 20, and the corresponding *P* values are computed using Wilcoxon rank sum test. Table 2 also reflect the maximum median values for each 19 electrodes using MSE, and the minimum *P* values are reflected from scale 1 to 20.

**Fig. 3:**
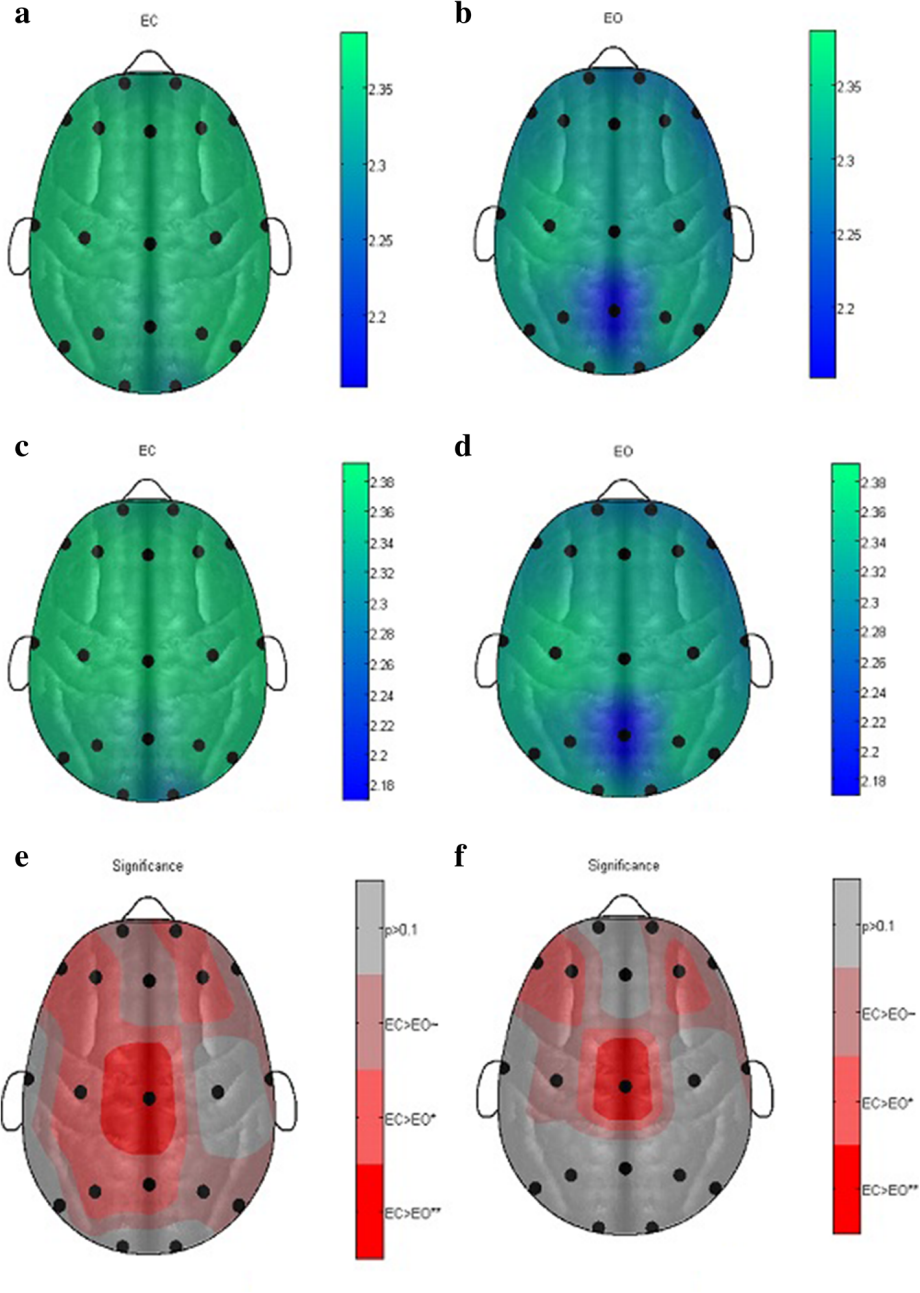
Topographic map representation of 19 electrodes according to standard 10–20 system with median MSE values. **a** EEG eye-closed subject during a resting state at scale 6. **b** EEG eye-open subject during a resting state at scale 6. **c** EEG eye-closed subject during a resting state at scale 7. **d** EEG eye-open subject during a resting state at scale 7. **e** EEG paired-wised significance EC vs EO at scale 6. **f** EEG paired-wised significance EC vs EO at scale 7

The performance of threshold-based symbolic entropy was also evaluated for distinguishing EC from EO during resting-state conditions on 14 selected electrode, and the results were compared to MSE. The results revealed that symbolic time series gives higher significant results than MSE at all electrodes except Fp2 which does not show any significant result as depicted in Table 2 and Figs. 2 and 3 in the form of topographic maps, whereas MSE shows the significance results in few electrodes only. The significant results using symbolic time series analysis were obtained at frontal electrodes F4 (*P* value 0.00043) and F7 (*P* value 0.00049); occipital region O1 (*P* value 0.00013) and O2 (*P* value 0.00043); and parietal region P7 (*P* value 0.00032) and P8 (*P* value 0.00085). While high significant results were obtained at electrodes C3 (*P* value 0.00704), F3 (*P* value 0.00143), F8 (*P* value 0.007), Fz (*P* value 0.00273), P3 and P4 (*P* value 0.0043) and T8 (*P* value 0.00983). Moreover, the electrodes C4, Cz, Pz and T8 gives only significant results. In comparison, the MSE gives only significant result to distinguish the EC from EO conditions during resting states at few electrodes such as C3, Cz, F3, F4, F7, O1, O2, P4, Pz, T8 and P8 only, and the other electrodes do not show any significant result.

In Table 2, we have compared the symbolic time series and MSE for which the minimum *P* values are reflected against each 19 electrodes. Symbolic entropy showed statistically significant results for 18 electrodes except Fp2 whereas MSE has not showed significant results at electrodes C4, F8, Fp1, Fp2, Fz, P3, T7 and P7. Moreover, the results showed that *P* values using symbolic time series analysis are lower than those using the MSE analysis method which is consistent with previous studies [31, 38, 47].

The NCSE strength is reflected on topographic maps which show the regions where the maximum NCSE values are obtained in the form of colourmaps. The colour scales reflect the strength of the NCSE values on each figure. Table 2 reflects only the maximum median values whereas the topographic maps reflect the median NCSE values at thresholds 1.2 and 1.4 where the maximum separation was obtained. The topographic maps also help to understand visually where the maximum strength of NCSE is obtained in the brain region. Thus, entropic measures can be best reflected in the form of topographic maps to investigate the overall underlying dynamics in a system.

Thus from the topographic maps in Fig. 3 (e), it can be seen that at threshold 1.2 and word length 3, the electrodes F7, F3 and P8 exhibits strictly significant results; electrodes Fz, F4, F8, T7, T8, C3, C4, Cz, P3, P4, P7, O1 and O2 shows very significant results where electrodes Fp1 and Pz only shows just significant results. Figure 3 (f) shows the statistically significant results in the form of topographic maps at threshold 1.4 and word length 3. In this case the electrodes F7, F3, F4, F8, T7, P7, O1, P4, and P8 exhibit strictly statistically significant results. The electrodes Fz, C3, Cz, T8, P3 and O2 also shows very significant results and Fp1, C4 and Pz shows just significant results.

**Fig. 4:**
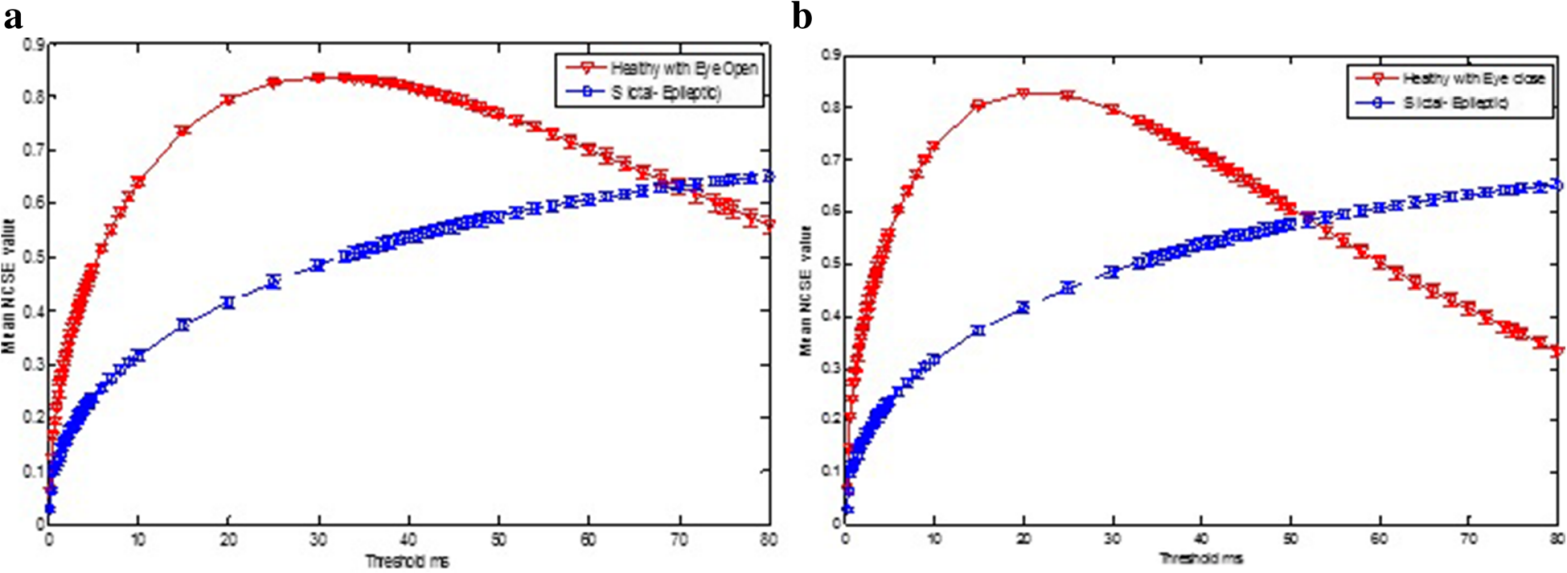
NCSE at different thresholds. **a** Healthy (set O) vs epileptic (set S). **b** Healthy (set Z) vs epileptic (Set S)

In the topographic maps in Fig. 4e, the overall MSE values of EC are found greater than those of EO and only Cz electrodes gives very significant result, whereas the electrodes F7, F3, F4, Fp2, C3, P3 and Pz were just found to be statistically significant. Moreover, the electrodes Fp1, Fz, P4, P8 and T8 are found almost significant, and T7, C4, P7, O1 and O2 exhibit no significance at all. In the topographic maps in Fig. 4f, again, only Cz gives very significant statistical results. Electrodes F7, F3 and F4 exhibit just significant results. and C3, Fp2, F8 and T8 are almost found significant. A larger number of electrodes as Fp1, Fz, T7, C4, P7, P8, P3, P4, Pz, O1 and O2 did not show any significance at all.

**Fig. 5:**
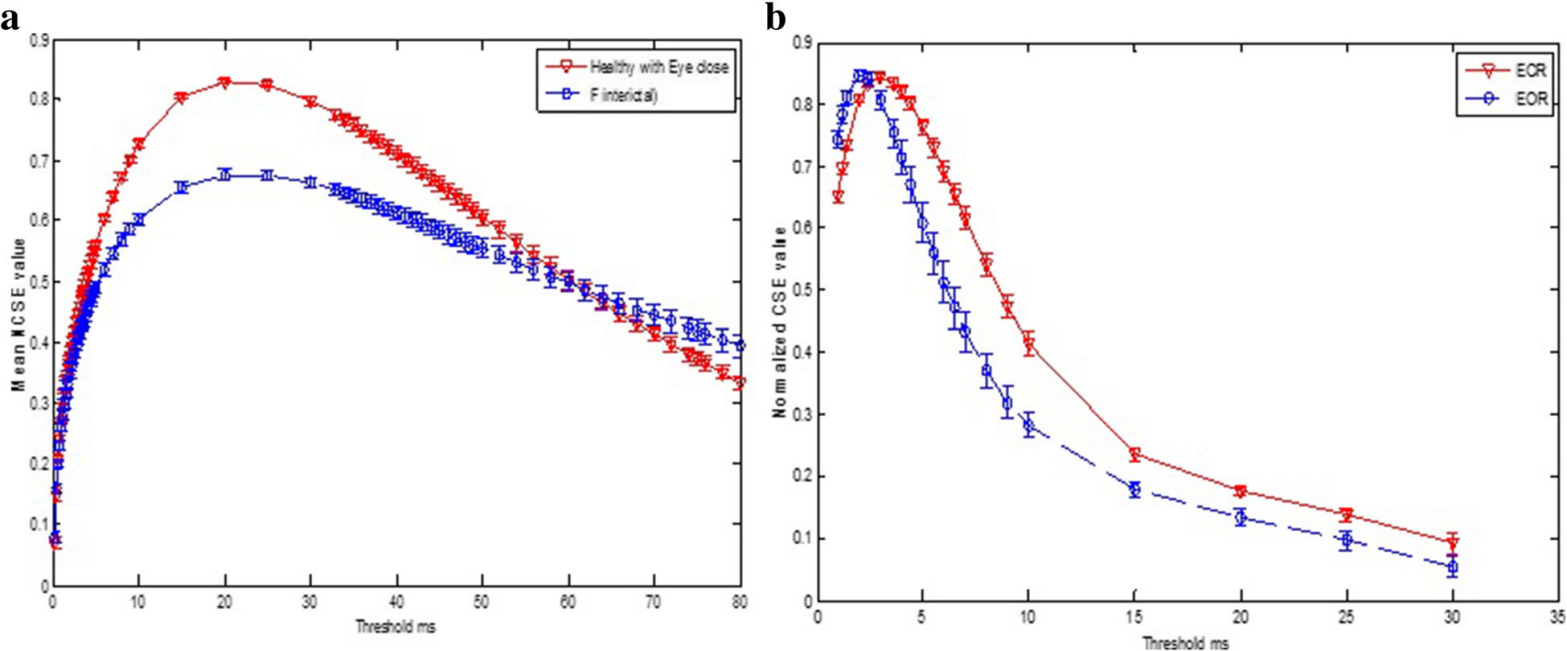
NCSE at different thresholds. **a** Healthy (set Z) vs epileptic seizure-free (set F). **b** EEG eye-closed vs EEG eye-open during a resting state

Figure 4a, b shows the comparisons of the NCSE values of the healthy subjects (with eye-open set O and eye-close set Z) with epileptic seizure subject (ictal interval) with an error bar. From the results, it is evident that the healthy subjects in both cases exhibit higher complexity than the epileptic subjects at certain threshold values. Figure 5a shows the comparison of the healthy subjects (eye-closed condition) to the epileptic seizure-free (interictal interval) subject and also depicts the higher NCSE values of the healthy subject than those of the interictal interval at a certain threshold. Figures 4 and 5 and Tables 1 also reflect that the epileptic seizure-free (interictal) intervals (both focal and non-focal) have also higher complexity values than the epileptic seizure (ictal) subject. In summary, it is clearly observed that the healthy subjects in both conditions, i.e. eye-open and eye-closed condition, exhibits higher complexity than the epileptic seizure and seizure-free intervals, whereas the seizure-free groups show higher complexity than the epileptic seizure (ict intervals) subjects at a certain threshold value. Figure 5b also shows the comparisons of the NCSE values of eye-closed and eye-open subjects during resting states at occipital electrode (O1). From Fig. 5b, it can be clearly depicted that the EO subjects shows greater NCSE values than the EC during resting states at certain smaller thresholds. Thus, EO is more complex than EC at these smaller threshold ranges.

Figure 4a, b shows the mean NCSE distribution of the healthy subjects with the eyes open (set O) vs the epileptic with seizures (ictal interval), the healthy with the eyes closed (set Z) vs the epileptic with seizures (ictal interval), the healthy with the eyes closed (set Z) and epileptic seizure-free interval (interictal set focal) and EC and EO during resting states. In all the cases, the symbols are used to denote the means, and bars to denote the standard error. The standard error is calculated using the formula Standard error = SD/√ *n* where *n* is the number of subjects in each case and SD denote the standard deviation. The most significant differences are seen at threshold 30 ms for set O with mean ± SD = 0.84 ± 0.00, sets Z, F and N at a threshold of 25 ms with mean ± SD of 0.83 ± 0.00, 0.68 ± 0.01 and 0.69 ± 0.01, respectively, whereas the mean ± SD for the epileptic seizure subject increased till threshold of 80 ms.

## Discussions and conclusions

The physiological system is composed of various subsystems, which are controlling the regularity mechanism of that system [33–36]. If all the subsystems are functional and working properly, the controlling mechanism of the overall system will be appropriate and the system is healthy. The healthy systems evolve with time and their adaptive capability is higher, resulting in higher complexity. The alternations in the structural components and/or decreased functional capability of the subsystem cause dysfunction in the regularity mechanism of the overall system, which results in the loss of complexity [33–36]. Various entropy-based methods have been proposed by researchers [33–38], each having its advantages and disadvantages in quantifying the dynamics of physiological system in healthy and disease. In this study, we have used threshold-based symbolic entropy to distinguish epileptic and healthy subjects as well as eye-open and eye-closed conditions. The results of the study were compared to those of the MSE [33], which has been used in several neurological studies to differentiate hypothermia and normothermia conditions [39], describe dynamical alternation owing to Alzheimer disease and dynamics of seizure-free, pre-seizure and during seizure states [41].

The NCSE values of the healthy subjects were higher than the epileptic subjects (including both ictal and interictal intervals) at wide range threshold values. Higher NCSE values for the healthy subjects at a wide range of thresholds manifest that probability of distribution of patterns (symbols sequence) is more uniform (almost equal chance of occurrence of all possible patterns). The higher probability of occurrence of all patterns (higher NCSE) reflects that the complexity of the healthy subjects higher. The loss of structural components and/or decreased functional capability of the subsystem due to ageing or disease perturbs the normal functioning of the overall system [36]. This perturbation may affect the probability of occurrence of all possible patterns which results in non-uniform distribution of pattern [35]. The decrease in uniformity of patterns due to dominancy of some patterns and/or non-occurrence of some pattern will result in low NCSE values. Thus, reduction in the NCSE values for epileptic subjects reflects the loss complexity, which is in line with the hypothesis ‘loss of complexity is a generic feature of pathological systems’ [33–36]. The MSE results also showed that the dynamics of healthy subjects are more complex compared to epileptic subjects; however, the results of NCSE are statistically more significant in distinguishing healthy and epileptic subjects. The one more advantage of using symbolic entropy measure is that it is robust short time series data, whereas MSE provides dynamically incorrect information at large temporal scales and may induce undefined entropy estimates.

The human brain displays five different waves to cope different situations, and each have a specific frequency band. The *δ* (0.5−4 Hz) wave activity is predominant in infants and occurs during deep sleep. *θ* (4−7.5 Hz) is found in normal awake adults but more prevalent during drowsiness. *α* (7.5−14 Hz) activity is attenuated by attention and found in the posterior head region in individuals who are relaxed, are awake and relaxed, and have their eyes closed. *β* (14−22 Hz) activity is associated with active attention and thinking by focusing outside the world or solving special types of problems usually the waking rhythm whereas γ (22−80 Hz) activities have very low amplitude and occur often. Alpha activity is dominant in normal individuals during resting states and is suppressed by visual stimulation [22–26]. Any brain wave either overproduced and/or underproduced can cause a problem. In this study, we also investigated changes in the dynamics of EO and EC conditions during resting states. The results indicated EO dynamics are more complex compared to EC dynamics. The resting state involves the different brain oscillations and particularly the alpha rhythm. The EO condition reflects increased visual system activity due to visual stimulation, probably mediated by the reticular activating system [27, 29, 58]. The increased visual activity may involve more structural components and hence escalate the coupling between additional functional resulting in more complex dynamics.

### Limitations of study and future recommendations

The research reported in this manuscript is focused on quantifying the dynamics of EEG signals during ictal and interictal intervals and to distinguish eye-closed condition from eye-open condition during resting states. The study revealed very interesting results; however, there are several limitations of this study such as the number of subjects is small and the lack of ageing and gender base analysis. Furthermore, dataset were taken from publicly available databases, and clinical profile of data was not available. Future studies can be designed to quantify the dynamics for the larger groups based on gender and age as well as to assess the suitability threshold symbolic for specific type of patients with epilepsy.
